# Editorial: Life skills in school health promotion: challenges and solutions

**DOI:** 10.3389/fpubh.2026.1950818

**Published:** 2026-08-24

**Authors:** Graça S. Carvalho, Teresa Vilaça, Carlos Alberto de Oliveira Magalhães-Júnior, Adeline Darlington-Bernard

**Affiliations:** 1 Research Centre for Child Studies, University of Minho, Braga, Portugal; 2 State University of Maringá, Maringá, PR, Brazil; 3 Claude Bernard Lyon 1 University, Lyon, France

**Keywords:** emotional competencies, health promotion, life skills, mental health, physical activity, school health, wellbeing

This Research Topic on “*Life skills in school health promotion: challenges and solutions*” aims to address the ongoing challenges in defining and understanding life skills within school health promotion and beyond. By deepening the current literature and engaging with health education and promotion professionals, it seeks to identify and resolve the conceptual ambiguities that hinder a unified comprehension of life skills. The ultimate objective is to propose more precise and applicable definitions and frameworks that can be embraced by health promotion policies and programmes globally. This endeavor will provide clarity and direction for policymakers, educators, and stakeholders striving to enhance health outcomes and the development of life skills among children, young people, adults and older people. A total of 41 manuscripts were submitted for consideration. Following a rigorous peer-review process and revisions based on expert feedback, 25 (60.1%) were accepted for publication. Among these, 9 explore *health promotion and wellbeing*, 8 examine *mental health and emotional competencies*, and 8 address *physical activity*.

## Health promotion and wellbeing

The original concept of health promotion refers to the process of enabling individuals and communities to increase control over the factors that influence their health and, consequently, to improve it (1). Wellbeing, in turn, encompasses a person's overall quality of life, including physical, mental, emotional, social, and spiritual dimensions (2). Health promotion extends beyond the healthcare sector, involving governments, educational institutions, workplaces, healthcare systems, families, and communities, rather than health professionals alone. Wellbeing, meanwhile, reflects the experience of living a healthy, meaningful, and fulfilling life (2).

Together, health promotion and wellbeing represent a holistic approach to health that empowers individuals and communities to enhance their health and quality of life by strengthening personal capacities, creating supportive environments, and addressing the social, economic, cultural, and environmental determinants of health. This approach aims to enable people to flourish physically, mentally, and socially while living in healthy and sustainable environments. Widely adopted in public health, education, and international policy, it reflects a paradigm shift from viewing health merely as the absence of disease to recognizing it as a positive resource for everyday life (Magalhães-Júnior et al.).

The nine articles included in the *Health Promotion and Wellbeing* Section address the following issues: Salque et al. analyzed the French Explo'Santé programme, a school-based health promotion intervention that combines structured health education sessions with supportive environments, showing that it supports the development of key psychosocial competencies, contributing to understanding how school-based health promotion can foster more equitable developmental trajectories; Wang, Li et al. reported that Chinese nursing interns' digital literacy and self-efficacy are moderate, requiring improved training in digital competencies with psychological empowerment strategies to enhance interns' skills in digital healthcare environments; Xu et al. observed that Chinese nursing students show low electronic health literacy skills to retrieve, evaluate, and apply digital health information in clinical practice and health education, recommending that hospitals should establish health consultation centers or virtual platforms to assist nursing students in identifying and evaluating online health information and addressing their practical health needs; Magalhães-Júnior et al. analyzed Portuguese children's social representations of coronavirus to design school health education strategies that must include scientific knowledge and develop critical analysis of social media disseminating information; Zhao et al. applied the Knowledge-Attitude-Behavior (KAB) model to Chinese college students, showing that Knowledge influenced Behavior directly and Attitudes indirectly, indicating that teaching disease prevention needs to be improved, particularly on hazards of antibiotics; Dumitru et al. adapted and validated the Standardized Patient Performance Rating Scale—Student Version (SPRS-S) for evaluating simulated patients during first aid training of school-aged students in Greece, the results of which showed three distinct performance clusters and indicated that this Greek version constitutes a clear and targeted assessment tool that is not affected by possible demographic differences; Ferreira Borges Paschoalini and da Silveira Paschoalini analyzed an experiential learning project where first-year medical students promoted menstrual health education within Brazil's school health programme for adolescents, showing that it provided a dual-purpose intervention, fostering socially accountable, humanistic competencies in future physicians while simultaneously advancing adolescent health literacy and dignity; Niu et al. used a self-developed scale to assess fertility awareness among Chinese university students, showing that the fertility awareness level of Chinese university students is generally low, and recommended healthcare institutions should implement targeted intervention measures to improve reproductive health outcomes; Zhuang et al. verified that Chinese female college students' trust in healthcare professionals affects price-related barriers and identified the high-risk profile “low trust and high price” as the dominant factor for HPV vaccination hesitancy.

## Mental health and emotional competencies

Mental health refers to an individual's capacity to realize their potential, cope effectively with life's stresses, work productively, and contribute meaningfully to their community (3). As a fundamental component of overall wellbeing, mental health encompasses a dynamic state of balance across cognitive, emotional, and behavioral domains (4). Within this context, social and emotional learning has become a central component of contemporary education, which encompasses the knowledge, attitudes, and life skills required to understand and regulate emotions, establish and achieve positive goals, develop healthy relationships, and make responsible decisions (5). These life skills are essential for healthy child development (Alkahtani et al.) and remain equally important during adolescence, a developmental period characterized by profound physical, psychological, and social changes that shape identity formation, foster increasing autonomy, and strengthen the acquisition of life skills (6).

Schools play a critical role in promoting mental health and wellbeing while addressing factors that threaten them. One such challenge is school bullying, a widespread phenomenon observed across diverse cultural contexts worldwide (7). School-based anti-bullying interventions seek to equip children and adolescents with the knowledge, skills, and coping strategies needed to prevent, respond to, and resist bullying behaviors (8).

Mental health challenges are not limited to childhood and adolescence but extend into adulthood. University and vocational students, particularly those enrolled in health-related programmes, are especially vulnerable due to demanding academic workloads, irregular class and clinical schedules, and the emotional and psychological pressures associated with providing patient care during their professional training (Wang, Ishak et al.; Abdulrahman et al.).

The eight articles included in the Section on *mental health and emotional competencies* address the following issues: Alkahtani et al. verified that a social and emotional learning (SEL) programme can effectively promote competencies in Saudi Arabian preschool children at risk of emotional and behavioral disorders, leading to recommendations for teacher training in SEL practices to improve communication with children and foster positive behaviors in the classroom; Al-ketbi applied a multi-method participatory approach for the development of a comprehensive school anti-bullying logic model in the Emirate of Abu Dhabi (UAE), offering a strategic roadmap for schools to effectively implement policies and programmes to minimize bullying and establish a safer, more supportive learning environment, emphasizing the need for concerted effort in solving intricate problems such as bullying to ensure that solutions are practical, relevant, and sustainable; Guevara-Cabello et al. explored how adolescents in a Peruvian public school perceive daily challenges, mobilize General Resistance Resources (GRRs), and exercise agency from a salutogenic perspective, showing that adolescents mobilized diverse GRRs (personal, social, spiritual, digital, and institutional) for emotional regulation, problem-solving, and meaning-making, although with gender effects; Liu et al. implemented training based on mindfulness among Chinese university students, who encounter culturally distinct stressors including academic pressure and familial expectations, demonstrating that mindfulness was negatively correlated with psychological distress and suggesting that the relationship between mindfulness and distress is contingent upon cultural factors; Wang, Mao et al. collaborators explored the mental health problems of Chinese higher vocational nursing students and their influencing factors and concluded that hospital, gender, liking for the nursing profession, thinking that the nursing profession was respected, feeling cared for during internship and self-efficacy were all significantly associated with the nursing students' mental health during internship; Wang, Ishak et al. conducted a study applying the Sidek Module Development Model (SMDM) to design and validate a multi-component lifestyle intervention module aimed at improving sleep quality among vocational nursing students in China, having validated it as scientifically robust, educationally feasible, and culturally responsive lifestyle designed to promote sleep health among nursing students; Mei et al. investigated the relationship between electronic device use time, sleep quality, psychological stress and self-rated health among Chinese medical students, providing insights to enhance health awareness and promote healthier behaviors; Bin Abdulrahman et al. developed a systematic review examining the proposals and consequences of stress management for students in medical education, acknowledging the challenges inherent to their academic journey and, from their analyses, they concluded that institutions can more effectively prepare their medical learners to overcome educational challenges and cultivate empathetic, resilient professionals skilled in delivering healthcare services.

## Physical activity

The World Health Organization's (WHO) *Guidelines on Physical Activity and Sedentary Behavior emphasize* that regular physical activity improves cardiopulmonary fitness, metabolic health, and mental wellbeing by reducing symptoms of anxiety and depression, thereby enhancing overall quality of life (9). Regular physical activity is also effective in preventing and managing non-communicable diseases (NCDs), including cardiovascular disease, stroke, diabetes, breast cancer, and colorectal cancer, while reducing the risk of hypertension, overweight, and obesity (10). Despite these well-established benefits, global participation in physical activity remains insufficient, with approximately 70% of the world's population failing to achieve the recommended minimum of 150 min of moderate-intensity physical activity per week (11). In response, the WHO has set a global target of reducing physical inactivity by 15% by 2030 (12).

The WHO's *Guidelines on Physical Activity and Sedentary Behavior* further recommend that children and adolescents aged 5–17 years engage in at least 60 minutes of moderate-to-vigorous physical activity daily throughout the week (13), including muscle- and bone-strengthening activities on at least 3 days per week (14). Accordingly, promoting regular physical activity during childhood and adolescence is widely recognized as a fundamental strategy for fostering healthy physical growth, psychological wellbeing, and lifelong healthy behaviors.

Similarly, concerns have emerged regarding the health of university students. National surveys have documented a sustained decline in physical fitness over the past two decades (15), accompanied by a persistent increase in mental health problems (16). These challenges are further intensified by growing academic demands, the transition to independent living, and increasing societal expectations (17). The rising prevalence of these interconnected physical and mental health concerns has reinforced the need for comprehensive, holistic educational approaches that integrate health promotion into higher education, with physical education playing a central role in supporting students' overall wellbeing (18).

The eight articles included in the *physical activity* section address the following issues: Watanabe et al., assuming that healthy life expectancy requires attention not only to physical fitness but also to the development of cognitive self-regulation skills during childhood, incorporated assessments of cognitive inhibitory control into early health education using the go/no-go task to assess inhibitory control alongside conventional physical fitness testing in children aged 3–14 years in Nagano (Japan), providing an enriched understanding of children's self-regulatory capacity; Kong et al. evaluated a family–school–community collaborative physical activity (PA) intervention on PA and exercise cognition in Chinese primary school students in a quasi-experimental study, showing that the intervention was associated with improvements in exercise cognition and perceived benefits, which supports the potential value of strengthening positive exercise perceptions within coordinated PA promotion efforts; Jing, Zhai et al. showed that exercise self-efficacy is associated with physical activity both directly and indirectly via behavioral intention, in vocational education students in Shanghai (China), also indicating that interventions enhancing self-efficacy and behavioral intention may support higher activity levels; Jia et al. investigated how combinations of factors lead to high physical activity levels among Chinese female university students, using methods grounded in Social Cognitive Theory (SCT), revealing the characteristics of “multiple conjunctural causation” and “causal asymmetry” in forming physical activity behaviors and complex compensatory and substitutive mechanisms among factors, moving beyond traditional linear models; Ma et al. explored the intensity characteristics of integrated “Learning, practice and competition” (L-P-C) badminton teaching in China, and showed that it primarily elicits moderate-intensity exercise, which is influenced by gender and skill levels, and improves cardiorespiratory fitness and psychological wellbeing; Wang, Feng et al. developed a policy-based Education Policy Dosing Index (EPDI, 0–100) from four exam-oriented domains (compulsory test intensity, elective test diversity, scoring/enforcement strictness, and competition & incentive provision) for the Chinese junior-middle-school PE examination scheme, showing that EPDI around 60 is a practical, exam-aligned planning benchmark, with diminishing marginal returns beyond the breakpoint and equity-relevant heterogeneity by sex and grade; Genç et al. demonstrated that Life Kinetik exercises effectively enhance motor coordination and cognitive performance in Turkish adolescents with intellectual disabilities (MID), more specifically, motor coordination and cognitive performance, suggesting that integrative exercise programmes may be a valuable non-pharmacological strategy to support motor and cognitive development in this population; Jing, Yang et al. employed a single-group pre-test/post-test design in a 21-day school-based regular aerobic exercise intervention in a comprehensive university in Sichuan (China), showing association with improvements in post-traumatic stress disorder (PTSD)-related symptoms and psychological resilience.

In summary, the 25 articles published in the Public Health Research Topic *Life skills in school health promotion: challenges and solutions* represent contributions from researchers across 14 countries spanning multiple continents. This broad international representation highlights the vitality and global relevance of this field of research. Collectively, these studies offer diverse approaches, perspectives, and methodological insights that enrich our understanding of life skills in health promotion. At the same time, they underscore the field's dynamic and evolving nature, revealing numerous opportunities for further multidisciplinary and cross-cultural research.
